# Retraction: Properly Folded Bacterially Expressed H1N1 Hemagglutinin Globular Head and Ectodomain Vaccines Protect Ferrets against H1N1 Pandemic Influenza Virus

**DOI:** 10.1371/journal.pone.0300096

**Published:** 2024-03-01

**Authors:** The PLOS ONE Editors

After publication of this article [1], concerns were raised about the geometric mean titers presented in Fig 3. Specifically, it was raised that the values presented in Fig 3 appear to be arithmetic means rather than geometric means as indicated in the Fig 3 legend.

During editorial follow up on this issue, the corresponding author stated that the figure is correct and the underlying data for Fig 3 is still available, but the data have not been provided to PLOS.

A member of the Editorial Board and a member of the Statistical Advisory Board both reviewed the concerns and comments provided and remain concerned with the reporting in Fig 3. The Editorial Board member stated that they do not believe the Fig 3 issue changes the conclusions of [1] as both arithmetic and geometric means are above the threshold of 1:40 that usually indicates an Ab response to vaccination. However, in following up on this issue, it was also noted that:

- Fig 2A displays individual values for all timepoints except for day 21, and for day 21 the graph does not appear to provide indication as to the variability of the results. Also, the sample size appears to be different for d0, d1-7, d14, and d21.
- The following results statement does not appear to correctly report the results in Fig 3: “The 30 μg dose induced 2–4 fold higher titers compared with the 7.5 μg dose for both bacterially expressed proteins”.
- The Fig 3 results cannot be interpreted without clarification on the variance representation.
- The graphs in Fig 4 do not indicate variance, and the article does not report statistical analyses to assess between-group differences for these results. Based on the reported information, it is difficult to draw conclusions about across-group differences. However, some results in the article do not appear to be supported:
  ○ The results statement about in vivo viral load on day 7 is not supported by Fig 4A, which only reports data through day 5.
  ○ For the graphs in Figs 4A-C, the sample size is small and there are concerns about the overall reliability of the claims about clinical outcomes in Figs 4B and 4C.
- In Tables 1 and 2, the mean (or geometric mean titer) of three replicates have been presented without standard deviation or range.

The authors did not comment on these issues.

In light of the unresolved questions about the statistical analyses, sample size for some experiments, and results statements that call into question the overall reliability of the article’s conclusions, the *PLOS ONE* Editors retract this article.

HG did not respond to the editorial decision. SK, SV, NV, CJC, DMC, JM, LRK and TMR either did not respond directly or could not be reached.

## References

[1] Khurana S, Verma S, Verma N, Crevar CJ, Carter DM, Manischewitz J, et al. (2010) Properly Folded Bacterially Expressed H1N1 Hemagglutinin Globular Head and Ectodomain Vaccines Protect Ferrets against H1N1 Pandemic Influenza Virus. PLoS ONE 5(7): e11548. 10.1371/journal.pone.0011548 20634959 PMC2902520

